# Assessment of the potency and effectiveness of a heptavalent oil-adjuvanted (ISA 206) foot-and-mouth disease vaccine in Egypt

**DOI:** 10.1007/s00705-022-05624-2

**Published:** 2023-01-12

**Authors:** Abdel-Hamid Bazid, Haitham M. Amer, Mohamed Nayel, Mohamed Attia, Nada Maklad, Momtaz Wasfy, Mohamed Abdelmegeid, Magdy M. El-Sayed, Asmaa Magouz, Yassien Badr

**Affiliations:** 1 Department of Virology, Faculty of Veterinary Medicine, University of Sadat City, Sadat City, 32897 Menoufia Egypt; 2 Department of Virology, Faculty of Veterinary Medicine, Cairo University, Giza, 12211 Egypt; 3 Department of Medicine and Infectious diseases, Faculty of Veterinary Medicine, University of Sadat City, Sadat City, Egypt; 4 Middle East for Veterinary vaccines, Second Industrial Area, El-Salhya El-Gedida, El-sharqia, Egypt; 5 Department of Animal Medicine, Faculty of Veterinary Medicine, Kaferelsheikh University, Kafr El-Sheikh, Egypt; 6 Department of Virology, Faculty of Veterinary Medicine, Kafrelsheikh University, Kafr El-Sheikh, Egypt; 7 Department of Animal Medicine, Faculty of Veterinary Medicine, Damanhour University, Damanhour, Egypt

## Abstract

Foot-and-mouth disease (FMD) is a serious highly contagious viral disease affecting all cloven-hoofed animals, and outbreaks can have a severe economic impact. An inactivated heptavalent oil-adjuvanted FMD vaccine (Aphtovac-7, MEVAC) was prepared from the foot-and-mouth disease virus (FMDV) strains A-Iran05, A-Africa-IV, O-PanAsia2, O-Manisa, O-EA3, SAT-2 Gharbia, and SAT-2 LIB-12. The vaccine potency and effectiveness were evaluated in three groups of 6- to 8-month-old calves and 200 adult dairy cattle under field conditions. All animals were vaccinated with the vaccine preparation, and the three groups of calves were challenged after 28 days by intradermolingual inoculation with 10^4^ 50% tissue culture infective dose (TCID_50_) of FMDV serotype A, O, or SAT-2. Mock-vaccinated calves (two per group) served as unvaccinated controls during the challenge test. Adult dairy cattle were tested for seroconversion using a virus neutralization test at 30, 60, and 120 days post-vaccination. All calves displayed complete protection against challenge with the different serotypes of FMDV when compared to the control groups. Serum samples collected after the primary and booster immunizations at 30 days post-vaccination contained high titers of protective antibodies (≥ 1/32; i.e. 1.5 log_10_). Antibodies persisted until the end of the study period (120 days), with a peak value around 60 days post-vaccination. The heptavalent FMD vaccine preparation was found to be potent and capable of providing a protective immune response under both experimental and field conditions.

## Introduction

Foot-and-mouth disease (FMD) is an acute highly contagious viral disease of cloven-hoofed animals with a great economic impact, mainly due to losses in body weight and milk yield, fatality in young animals, and abortions in pregnant animals [1, 2]. Foot-and-mouth disease virus (FMDV) is a member of the genus *Aphthovirus* of the family *Picornaviridae* and has seven distinct serotypes (A, C, O, Asia 1, Southern African Territories [SAT] 1, SAT2, and SAT3). The high genetic and antigenic variability of FMDV and the existence of many lineages and sublineages are mainly due to error-prone RNA replication and extensive intra- and interserotype recombination [3, 4]. Based on VP1 gene sequences, the serotypes are further classified into antigenically different topotypes [5]. Protective immune responses are elicited either by vaccination or by natural infection with a homologous strain [6]. Both Asian and African FMDV strains have been found in Egypt due to the unique transcontinental geographical location of the country. Serotypes A, O, and SAT2 have been co-circulating in Egypt since 2013. However, recent molecular studies have revealed the existence of numerous genotypes, topotypes, and lineages within these serotypes among field isolates in Egypt [7, 8]. Hence, it is necessary to update the current vaccines with the predominantly circulating strains in order to ensure optimal protection.

Many attempts have been made to enhance the immunogenicity and potency of inactivated vaccines [6]. The current FMDV status in Egypt has led to the formulation of FMD vaccines that include at least five strains. These strains represent lineages belonging to the five topotypes reported during the last 10 years, including one of serotype SAT2, two of serotype A, and three of serotype O. Such multivalent vaccines have been formulated recently by some vaccine manufacturers [9].

The vaccinal strain and the type of the adjuvant are the most crucial factors that influence the immunogenicity of FMD vaccines. Adjuvants provide an excellent means of improving the efficiency of FMD vaccines by potentiating immune responses [10]. For inactivated FMD vaccines, alum-based and mineral-oil-based adjuvants with or without saponin are frequently used [11]. However, vaccines containing aluminum hydroxide and saponin as adjuvants have many disadvantages, such as the tendency to induce a rapidly waning immune response, thus requiring revaccination at intervals of 4-6 months. In addition, vaccines with aluminum adjuvant may cause allergic reactions and neurotoxicity due to high levels of IgE induction [12]. Conversely, FMD vaccines with oil-based adjuvants can elicit high antibody titers and long-lived immune responses [13]. Moreover, oil-based adjuvant vaccines can overcome interference by maternal antibodies in neonates and can therefore be administered to younger calves, in contrast to alum-based adjuvant vaccines [14]. Montanide ISA 206 VG is a mineral-oil-based adjuvant manufactured by SEPPIC, France, and is used to formulate water-in-oil-in-water (W/O/W) emulsions that are safe, stable, easily injected due to their low viscosity, and the absence of adverse effects, pyrogenicity, or granulomas. They are also more appropriate for antigens with low immunogenicity [15]. In the present study, the potency and effectiveness of a heptavalent oil-based FMD vaccine (Aphthovac-7, MEVAC^®^) containing the FMDV strains/serotypes A-Iran05, A-Africa-IV, O-PanAsia2, O-Manisa, O-EA3, SAT-2 Gharbia and SAT-2 LIB-12 mixed with Montanide ISA 206 VG were evaluated. Although no major scientific breakthroughs were reported, the study provides detailed information on the field application and expected reactions of the inactivated heptavalent FMD vaccine.

## Materials and methods

### Animals

In the vaccine safety experiment, two calves aged 6 to 8 months that were clinically healthy and seronegative for FMDV received the vaccine (double the recommended dose) and were observed for any post-vaccination reaction. In the vaccine potency experiment, 21 calves aged 6 to 8 months that were clinically healthy and seronegative for FMDV, were monitored for antibody responses, and were used in the challenge experiments. In the vaccine effectiveness experiment, the vaccine was evaluated on a dairy farm containing 200 dairy cattle with no history of previous vaccination or exposure.

### FMDV strains

All virus work was conducted in biosafety level 3 laboratories at Middle East for Vaccines Company (MEVAC), Egypt. FMDV serotypes/strains (A/EGY/1/2010, A/EGY/1/A/2012, O/EGY/4/2012, O/Sharquia/EGY/2014, O1/Sharquia/EGY/72, SAT2/EGY-A-2012, and SAT2/EGY/2018) were propagated in baby hamster kidney strain 21 (BHK-21) suspension cells. Aseptically harvested FMDV was clarified using 1% chloroform to remove cell debris. The titers of the viruses were 7.3, 7.5, 7.6, 7.9, 6.3, 6.6, and 6.3 log_10_ 50% tissue culture infective dose (TCID_50_) for A/EGY/1/2010, A/EGY/1/A/2012, O/EGY/4/2012, O/Sharquia/EGY/2014, O1/Sharquia/EGY/72, SAT2/EGY-A-2012, and SAT2/EGY/2018) , respectively.

### Virus inactivation and concentration

FMDV was inactivated by two cycles of treatment with 3 mM binary ethylamine (BEI) [16]. Excess BEI was neutralized using sterile 6 mM sodium thiosulphate. The inactivated antigens were concentrated using a TFF filter (Consieve^®^ from Cobetter, catalog no. UFEFL0300050P) and eluted with Tris-KCl buffer pH 7.6 [17]. The 146S particles in the concentrated antigen preparations were analyzed by sucrose density gradient ultracentrifugation and by determining the absorbance at 254 nm using an ISCO 520C density gradient system [18].

### Antigen preparation and vaccine formulation

The aqueous phase of the seven serotypes/strains was prepared using different concentrations of 146S viral particles, depending on the stability and immunogenicity of each strain. The vaccine dose was 3-6 µg of each strain in a volume of 3 ml. The inactivated vaccine consisted of equal volumes of the oil phase (Montanide ISA 206VG, SEPPIC, France) and the aqueous phase, which were mixed thoroughly according to manufacturer’s instructions.

### Safety and sterility test

The vaccine preparation was tested for viral and bacterial sterility. The safety test was conducted to detect any abnormal local or systemic adverse reactions. Briefly, two healthy seronegative calves were inoculated via the subcutaneous (S/C) route with 6 ml (double the recommended dose) of the vaccine as recommended by the World Organization for Animal Health (WOAH) [19] and kept under observation for 10 days for the detection of local or systemic reactions.

### Potency test

Vaccine potency was estimated in experimentally vaccinated animals: directly by evaluating resistance to live virus challenge and indirectly by measuring specific antibody levels induced by vaccination using the virus neutralization test (VNT).

Protection against live virus challenge was tested using three virus topotypes (A-Africa [GIV], O-EA3, and SAT-2 [Lib12]), and the percentage of protection against generalized foot infection was estimated as described in the 2022 WOAH FMD manual [19]. Briefly, 21 native calves aged 6-8 months were used. These calves were clinically healthy and free of antibodies against FMDV, as determined by VNT. Fifteen calves were inoculated subcutaneously with 3 ml (the recommended dose) of the vaccine preparation. The other six calves were injected by the same route with adjuvant alone as controls. At 28 days post-vaccination, the 15 vaccinated calves were divided into three groups (five calves/group), and each group were challenged by intradermolingual inoculation with one of the virulent homologous FMDV topotypes at a dose of 10^4^ TCID_50_ per animal. The six control calves were also divided into three groups (two calves/group) and challenged by the same way as the vaccinated calves.  For indirect evaluation of vaccine potency, two serum samples were collected from all calves, one just before vaccination, and one at 28 days post-vaccination, and tested by VNT.

### Evaluation of vaccine effectiveness under field conditions

The newly formulated vaccine (Aphthovac-7) was used under field conditions to inoculate 200 animals on a dairy farm, which received two doses (a primary dose and a booster dose) one month apart. Fifty serum samples were collected at 30, 60, and 120 days post-vaccination for evaluation of seroconversion by VNT.

### Virus neutralizing antibody assay

VNT was carried out for the quantitative estimation of neutralizing antibodies against FMDV using sera collected 28 days after experimental vaccination for vaccine potency evaluation. Samples were also collected just before vaccination for comparative purposes. Similar VNT assays were conducted on sera from vaccinated cattle under field conditions for evaluation of vaccine effectiveness (WOAH manual 2022 [19]). For VNT, sera were filtered and heat inactivated in a water bath at 56°C for 30 minutes before testing, and twofold serial dilutions were then prepared from 1:4 to 1:512 in flat-bottomed microtiter plates (three wells/dilution). A volume of 50 µl of tissue culture fluid containing an estimated 100 TCID_50_ (with an accepted range from 32 to 320 TCID_50_) of FMDV was added to each serum dilution, and the plate was incubated for 1 hour at 37°C. Then, 50 µl of BHK-21 cell suspension at approximately 10^6^ cells/ml in a medium containing 10% specific-antibody-negative fetal calf serum was added to each well. Positive, negative, and medium controls and a standard antiserum of known titer were tested in each plate. The cells were incubated for two days at 37°C with 3% CO_2_ and observed for a cytopathic effect. On the third day, the plates were fixed with 10% formol/saline for 30 minutes and stained by immersion in 0.05% methylene blue in 10% formalin for 30 minutes and then rinsed with tap water. Finally, antibody titers were expressed as the reciprocal of the final dilution of serum required to neutralize 100 TCID_50_ of the virus. The test was valid when the positive standard serum was within a twofold dilution of the expected titer when the amount of virus per well was in the range of log_10_ 1.5–2.5 TCID_50_.

### Statistical analysis

Data were organized into tables and figures using Microsoft Excel 2016. A normality test was applied to determine whether the data were parametric or non-parametric, using the Shapiro-Wilk test at a significance level of 0.05. Differences between time points were evaluated using Friedman’s test at a significance level of 0.05. Differences between groups were evaluated using the Kruskal-Wallis test, and the overall effect was evaluated using two-way ANOVA. Statistical analysis was performed using IBM SPSS ver. 28 for Mac OS.

## Results

### Safety of the FMD vaccine preparation

The inoculated calves were observed for 10 days after inoculation. Rectal body temperatures ranged from 38.2 to 38.7 ºC. No negative side effects were observed after vaccination. In some calves, localized inflammatory swelling was observed at the site of injection (Table 1).

**Table 1:** *In vivo* safety of the FMD vaccine preparation

Days post-injection	Calf 1		Calf 2	
	Rectal temp. (°C)	Injection site	Rectal temp. (°C)	Injection site
1	38.2	_	38.3	_
2	38.5	_	38.6	S
3	38.5	S*	38.6	S
4	38.6	S	38.7	S
5	38.3	S	38.6	S
6	38.4	S	38.4	S
7	38.2	_	38.3	S
8	38.2	_	38.3	S
9	38.2	_	38.2	_
10	38.2	_	38.2	_

*S: inflammatory swelling

### Challenge test of the heptavalent vaccine in cattle

The topotypes A-Africa (GIV), O-EA3, and SAT-2 (Lib12) were used to challenge the vaccinated animals. Lesions on the tongue were observed in one, two, and three of the five animals in the respective challenge groups (Table 2). No foot lesions were detected in any of the vaccinated animals after challenge (Table 2). No substantial change in body temperature was observed that could be considered indicative of fever.

**Table 2:** Results of challenge of heptavalent-vaccine-inoculated cattle with the virus strains A-Africa (GIV), O-EA3, and SAT-2 (Lib12)

Serotype	Number of animals	VNT^a^ titer (log_10_)		Lesions		Protection against generalization (%)
		0 DPV	28 DPV^b^	Tongue	Feet	
A-Africa (GIV)	5	0.20	1.8	1/5	0	100%
O-EA3	5	0.00	1.7	2/5	0	100%
SAT-2 (Lib12)	5	0.00	1.6	3/5	0	100%
Control A-Africa (GIV)	2	0.00	0.00	2/2	2/2	0.0%
Control O EA3	2	0.00	0.00	2/2	2/2	0.0%
Control SAT-2 (Lib-12)	2	0.00	0.00	2/2	2/2	0.0%

^a^VNT: virus neutralization test

^b^DPV: days post-vaccination

### Serological responses

**Fig. 1:**
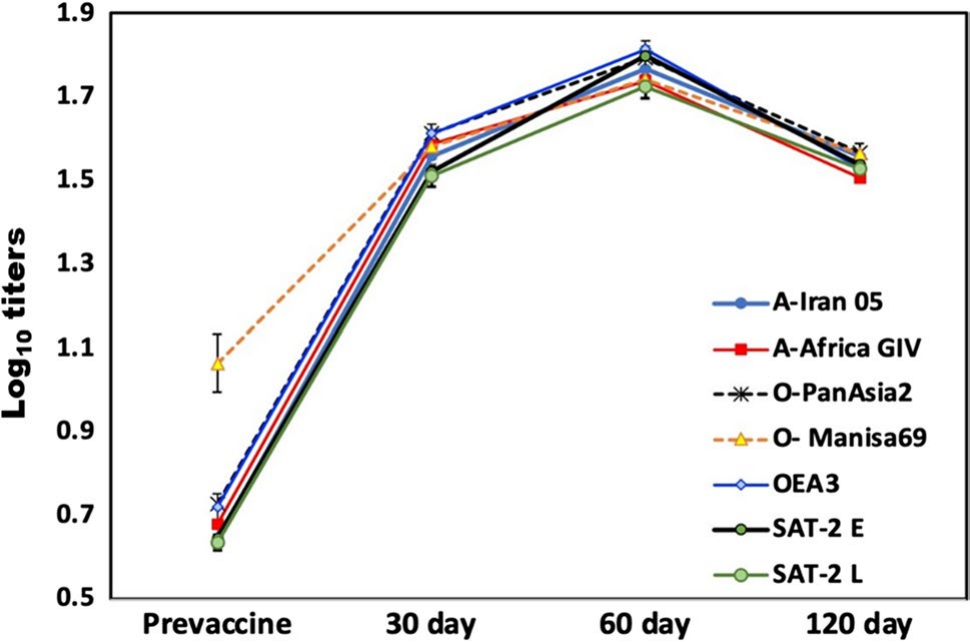
Changes in the mean antibody titers estimated at different days (0, 30, 60, and 120) post-vaccination in cattle vaccinated with the heptavalent vaccine

Serum samples collected from dairy cattle before vaccination had low FMDV antibody titers (0.6-0.9 log_10_), but after the primary and booster immunizations, they showed protective antibody levels at 30 days post-vaccination (VNT: 1.57 ± 0.16, 1.60 ± 0.2, and 1.52 ± 0.17 against serotypes A, O, and SAT-2, respectively; Fig. 1). Antibodies persisted until the end of the experiment (120 days), with a peak value around 60 days post-vaccination (VNT: 1.75 ± 0.18, 1.78 ± 0.17, and 1.76 ± 0.21 for serotypes A, O, and SAT-2, respectively; Fig. 1). Data are expressed as the mean ± standard deviation.

According to two-way analysis of variance, a highly significant difference between serotypes was observed (F = 10.9; *p* < 0.001), as well as a highly significant effects of time (days) (F = 1906.4; *p* < 0.001) and interaction between serotypes and time (F = 7.7, *p* < 0.001) were observed. Differences between serotypes within different time points, including pre-vaccination, 30 days, and 60 days, showed a significant difference in antibody titers (Table 3). However, at 120 days, a non-significant difference between serotypes was observed, using the Kruskal-Wallis test. For further comparisons between pairs of serotypes, Dunn’s Bonferroni test was performed, and means followed by different letters in the same column in Table 3 are significantly different at the 0.05 level. The change in antibody titer for each serotype with time showed a highly significant difference in all strains, as revealed by Friedman’s test for related samples (Table 3).

**Table 3:** The mean antibody titers estimated pre-vaccination and at different days (30, 60, and 120) post-vaccination in cattle vaccinated with the heptavalent vaccine

Serotype	Pre-vaccination			30 days			60 days			120 days			Correl (r)	Friedman’s test
	Mean	SD	Dunn’s	Mean	SD	Dunn’s	Mean	SD	Dunn’s	Mean	SD	Dunn’s		
A-Iran 05	0.64	0.15	b	1.56	0.17	abc	1.77	0.16	abc	1.56	0.18	A	0.596***	<0.001***
A-Africa GIV	0.68	0.23	b	1.59	0.12	ab	1.74	0.16	bc	1.51	0.15	A	0.488***	<0.001***
O-PanAsia2	0.73	0.32	b	1.61	0.17	a	1.79	0.16	abc	1.57	0.20	A	0.488***	<0.001***
O- Manisa69	1.06	0.49	a	1.58	0.21	ab	1.74	0.14	bc	1.56	0.18	A	0.321***	<0.001***
OEA3	0.72	0.23	b	1.61	0.17	a	1.81	0.15	a	1.53	0.17	A	0.516***	<0.001***
SAT-2 E	0.65	0.16	b	1.52	0.13	bc	1.80	0.18	ab	1.54	0.15	A	0.624***	<0.001***
SAT-2 L	0.64	0.13	b	1.51	0.17	c	1.72	0.20	c	1.53	0.11	A	0.588***	<0.001***
Krusksal-Wallis	< 0.001***			0.005**			0.044*			0.486 ns				

Two-way ANOVA	F	*p*-value	Partial eta squared
Corrected model	219.4	< 0.001***	0.812
Serotype	10.9	< 0.001***	0.045
Time	1906.4	< 0.001***	0.807
Serotype * Time	7.7	< 0.001***	0.092

*, **, ***, significant at *p* < 0.05, < 0.01, and < 0.001, respectively; ns, non-significant at *p* > 0.05

a, b, c: means followed by different letters are significantly different vertically (in the same column).

## Discussion

In Egypt, FMD is an enzootic disease, with outbreaks recurring at frequent intervals despite the use of various commercial vaccines [20]. Many of the current strains in Egypt were introduced by the annual importation of livestock from countries such as Sudan, Somalia, and Ethiopia [20]. Egypt is attempting to control and finally eradicate FMD through mass immunization campaigns. Along with compulsory vaccination, strict biosecurity precautions and FMD monitoring are being implemented. The introduction of the FMD control strategy has led to a significant reduction in FMD cases, but outbreaks are still being reported, even in areas where immunization is done on a regular basis. Declining herd immunity, poor animal health, FMD carrier status, and FMD transmission from other species are all factors that contribute to vaccine failure [1].

Inactivated vaccines commonly have poor immunogenicity, requiring the addition of appropriate adjuvants to increase the immune response to the antigens included. Furthermore, adjuvants may minimize the antigen dose or number of immunizations required to elicit a protective immune response, resulting in increased vaccine availability and lower vaccine costs [6, 21]. Montanide ISA-206 is a mineral-oil-based adjuvant that contains octadecenoic acid esters and anhydromannitol in an oily solution. It is added to the FMDV antigens to create FMD w/o/w emulsion vaccines used in several countries of the world [22]. Compared to Al(OH)_3_-adjuvanted vaccines, oil-adjuvanted formulations produce stronger and longer-lasting immune responses [23]. In cattle, FMDV-specific antibody responses were recorded for at least 92–120 days after vaccination [24]. Published studies have reported that ISA-206 formulations are superior to Al(OH)_3_- or saponin-based formulations and that they provide longer protection [25]. ISA-206-based emergency vaccinations have also been shown to protect cattle as early as two days post-vaccination [6]. In our study, no negative side effects and a few cases of rapidly resolving localized inflammatory swelling at the injection site were observed, demonstrating that the antigen/adjuvant combination is safe for use in cattle. In addition, Montanide ISA-201(206) was found to improve the efficiency of induction of neutralizing antibodies against FMDV, as has been reported by other researchers [26]*.* The mean neutralizing antibody titers in vaccinated cattle and the time at which they reached a maximum in our study was in agreement with the results of previous studies [6, 23]. These findings also support the superiority of Montanide ISA-206 as a mineral oil-based adjuvant to alum-based adjuvants [23], although this was not comparatively assessed in the current study. The lack of cross-protection between different FMDV serotypes, incomplete protection between some subtypes of FMDV, and the periodic emergence of new variant FMD viruses may affect the long-term application of a vaccine in the field. Consequently, regular vaccine strain selection by either *in vivo* or *in vitro* methods is a crucial requirement to enable the use of appropriate and efficient vaccines.

Animal challenge trials used to evaluate vaccine efficacy are mainly based on assessment of reduced clinical illness and viral shedding as measured by virus cultivation in cell culture or RT-PCR [27]. Potency testing involves assessing the protection rate in calves inoculated with a vaccine by challenge with a homologous virus [19]. The results of this study showed that a single administration of the heptavalent ISA 206 VG oil-adjuvanted vaccine resulted in a high mean neutralizing antibody titer 28 days post-vaccination. It corresponded to complete protection in vaccinated animals after challenge with 10^4^ TCID_50_ of each of the three homologous FMDV topotypes used in the vaccine. In our study, we used the protection against generalization (PG) method described by Vianna Filoh and colleagues [28]. The absence of foot lesions in vaccinated animals after challenge indicates protection from generalization of the disease (Table 2), while the control animals must have lesions on at least three feet. The results obtained in this study demonstrate that the performance of the examined vaccine was comparable to that of the current inactivated FMDV vaccines, being effective against generalized disease. Under field conditions, the heptavalent vaccine provided high neutralizing antibody titers after two doses (prime and booster doses), which continued for four months, indicating the vaccine's ability to provide clinical protection in the field.

The results of this study demonstrated the potency and effectiveness of a heptavalent FMD vaccine formulated with ISA 206 VG (W/O/W) emulsion oil-based adjuvant (Aphthovac-7, MEVAC^®^) containing enzootic virus strains/serotypes present in Egypt. The findings suggest that the inactivated vaccine could be effective in the control and mitigation of FMD, particularly in enzootic countries.

## Data Availability

Data supporting the findings are available on request from the authors.

## References

[1] Singh RK, Sharma GK, Mahajan S (2019). Foot and mouth disease virus: immunobiology, advances in vaccines and vaccination strategies addressing vaccine failures-an indian perspective. Vaccines.

[2] Jamal SM, Belsham GJ (2013). Foot-and-mouth disease: past, present and future. Vet Res.

[3] You SH, Jo HE, Choi JH (2019). Evaluation of novel inactivated vaccine for type C foot-and-mouth disease in cattle and pigs. Vet Microbiol.

[4] Jamal SM, Nazem Shirazi MH, Ozyoruk F (2020). Evidence for multiple recombination events within foot-and-mouth disease viruses circulating in West Eurasia. Transbound Emerg Dis.

[5] Abdulrahman DA, El-Deeb AH, Shafik NG, Shaheen MA, Hussein HA (2019). Mutations in foot and mouth disease virus types A and O isolated from vaccinated animals. Rev Sci Tech.

[6] Dar P, Kalaivanan R, Sied N, Mamo B (2013). Montanide ISA™ 201 adjuvanted FMD vaccine induces improved immune responses and protection in cattle. Vaccine.

[7] Ahmed ES, Hussein HA, Mahmoud NA (2016). Isolation and characterization of foot and mouth disease virus serotype SAT2 circulating in Egypt in 2014. Global Vet.

[8] Soltan MA, Negmaldin AH, El-Diasty MM (2017). Molecular characterization of circulating foot and mouth disease virus (FMDV) serotype O topotype EA-3 and serotype A (African topotype) genotype IV in Egypt, 2016. Vet Microbiol.

[9] Abu-Elnaga HI, Rizk SA, Daoud HM, Mohamed AA (2020). Comparative nucleotide sequencing of the VP1 capsid gene of recent isolates of foot-and-mouth disease virus serotype O from Egypt. Arch Virol.

[10] Petrovsky N, Aguilar JC (2004). Vaccine adjuvants: current state and future trends. Immunol Cell Biol.

[11] Park ME, Lee SY, Kim RH (2014). Enhanced immune responses of foot-and-mouth disease vaccine using new oil/gel adjuvant mixtures in pigs and goats. Vaccine.

[12] Ensen-Jarolim E (2015). Aluminium in Allergies and Allergen immunotherapy. World Allergy Organ J.

[13] Bazid AI, El-Alfy HA, El-Didamony G (2021). Adjuvant effect of saponin in an oil-based monovalent (serotype O) foot-and-mouth disease virus vaccine on the antibody response in guinea pigs and cattle. Arch Virol.

[14] Sadir AM, Schudel AA, Laporte O, Braun M, Margni RA (1988). Response to foot-and-mouth disease vaccines in newborn calves. Influence of age, colostral antibodies and adjuvants. Epidemiol Infect.

[15] SEPPIC (2010) Montanide ISA 61 VG. In S. Inc (ed) abbreviated Montanide ISA 61 VG. http://www.seppic.com/file/galleryelement/pj/7d/7b/48/55/4524-technicalbulletin-mtd-isa-61-vg3096961497520821468.pdf. Accessed: 15, March, 2022

[16] Barteling SJ, Cassim NI (2004). Very fast (and safe) inactivation of foot-and-mouth disease virus and enteroviruses by a combination of binary ethyleneimine and formaldehyde. Dev Biol.

[17] Barteling SJ, Meloen RH (1974). A simple method for the quantification of 140S particles of foot-and-mouth disease virus (FMDV). Arch Gesamte Virusforsch.

[18] Doel TR, Chong WK (1982). Comparative immunogenicity of 146S, 75S and 12S particles of foot-and-mouth disease virus. Arch Virol.

[19] WOAH (2022) Foot and Mouth Disease (infection with foot and mouth disease virus). In: Manual of Diagnostic Tests and Vaccines for Terrestrial Animals 2022, Chapter 3.1.8: World Organization for Animal Health (Previously: Office international des epizooties [OIE]), Paris, France

[20] Al-Hosary AA, Kandeil A, El-Taweel AN (2019). Co-infection with different serotypes of FMDV in vaccinated cattle in Southern Egypt. Virus Genes.

[21] Aguilar JC, Rodriguez EG (2007). Vaccine adjuvants revisited. Vaccine.

[22] Cao Y (2014). Adjuvants for foot-and-mouth disease virus vaccines: recent progress. Expert Rev Vaccines.

[23] Khorasani A, Madadgar O, Soleimanjahi H, Keyvanfar H, Mahravani H (2016). Evaluation of the efficacy of a new oil-based adjuvant ISA 61 VG FMD vaccine as a potential vaccine for cattle. Iran J Vet Res.

[24] Barnett PV, Carabin H (2002). A review of emergency foot-and-mouth disease (FMD) vaccines. Vaccine.

[25] Choi JH, You SH, Ko MK, Jo HE, Shin SH, Jo H, Lee MJ, Kim SM, Kim B, Lee JS, Park JH (2020). Improved immune responses and safety of foot-and-mouth disease vaccine containing immunostimulating components in pigs. J Vet Sci.

[26] Iyer A, Ghosh S, Singh S, Deshmukh R (2000). Evaluation of three ‘ready to formulate’ oil adjuvants for foot-and mouth disease vaccine production. Vaccine.

[27] Waters R, Ludi AB, Fowler VL, Wilsden G (2018). Efficacy of a high-potency multivalent foot-and-mouth disease virus vaccine in cattle against heterologous challenge with a field virus from the emerging A/ASIA/G-VII lineage. Vaccine.

[28] Vianna Filho YL, Astudillo V, Gomes I, Fernández G, Rozas CEE, Ravison JA, Alonso A (1993). Potency control of foot-and-mouth disease vaccine in cattle. Comparison of the 50% protective dose and the protection against generalization. Vaccine.

